# Reference Ranges of 2-Dimensional Placental Biometry and 3-Dimensional Placental Volume between 11 and 14 Weeks of Gestation

**DOI:** 10.3390/diagnostics14141556

**Published:** 2024-07-18

**Authors:** Cristina Trilla Solà, Juan Parra Roca, Elisa Llurba Olivé

**Affiliations:** 1 Department of Obstetrics and Gynecology, Institut d’Investigació Biomèdica Sant Pau-IIB Sant Pau, Hospital de la Santa Creu i Sant Pau, 08025 Barcelona, Spain; 2 Faculty of Medicine, Universitat Autònoma de Barcelona, 08025 Barcelona, Spain; 3 Primary Care Interventions to Prevent Maternal and Child Chronic Diseases of Perinatal and Developmental Origin Network (RICORS, RD21/0012/0001), Instituto de Salud Carlos III, 28040 Madrid, Spain

**Keywords:** first trimester, placental biometry, placental volume, 2-dimensional ultrasound, 3-dimensional ultrasound, preeclampsia, placental insufficiency

## Abstract

Objective: The purpose of this study was to provide gestational age (GA) specific reference ranges for 2-dimensional (2D) placental biometry and 3-dimensional (3D) placental volume between 11 and 14 weeks of gestation. Methods: Placental biometry including 2D and 3D variables was calculated in 1142 first-trimester singleton pregnancies with non-complicated outcome between September 2016 and February 2020. Ultrasound datasets were obtained at the time of the first-trimester ultrasound, and 2D basal plate (BP), chorionic plate (CP), placental thickness (PT), and 3D placental volume (PV) were measured following a standardized methodology. Reference ranges for each variable were calculated according to GA and crown-rump-length (CRL). Results: A total of 1142 uncomplicated pregnancies were considered for analysis. All placental measurements increased significantly between 11 and 14 weeks, especially for PT (39.64%) and PV (64.4%). Reference ranges were constructed for each 2D and 3D first-trimester placental variable using the best-fit regression model for the predicted mean and SD as a function of GA and CRL. Conclusions: Reference ranges of 2D placental biometry and 3D placental volume between 11 and 14 weeks of gestation were constructed, generating reference values. Placental biometry showed a progressive increase during the first trimester. This highlights the importance of using reference range charts according to GA.

## 1. Introduction

The placenta is a fundamental organ for fetal development [1,2]. This specialized organ is responsible for the exchange of nutrients, gases, and fetal metabolism products between the maternal and fetal bloodstreams, ensuring that the necessary resources are delivered to the fetus for proper growth and development [3]. Placental development begins with implantation of the embryo at the earliest stages of pregnancy [4,5]. By the end of the first trimester, the foundation of placental development is already established, although trophoblastic infiltration into the myometrium is progressive until 18 weeks of gestation [6]. The extent of trophoblastic invasion will determine subsequent placental efficiency and, consequently, fetal viability throughout gestation. Therefore, any alteration or disruption of placentation can have important implications for the health of both the mother and child [7,8]. Given the complexity of placental development and functioning, it is important to improve placental assessment in a comprehensive manner.

Several variables have been described to assess placental function throughout pregnancy including biochemical assessments and imaging techniques. Placental size has been associated with various obstetric complications such as preeclampsia, fetal growth retardation, placental abruption, congenital infection, or gestational diabetes [9,10]. It has also been established that placental size correlates well with neonatal weight at birth, and thus placental biometry has been suggested as a potential tool to identify pregnancies at higher risk for adverse obstetric outcomes [11].

Placental development and function can be evaluated using different imaging techniques [12,13,14]. Technological advances have allowed for the development of 3-dimensional (3D) ultrasound methodologies to estimate placental volume (PV) that have been proven to be reproducible and have good correlation with fetal birth weight and final placental weight [15,16,17]. Thus, placentas with smaller volumes in the first or second trimester are associated with infants and placentas with lower birth weight [10]. Other placental variables have also been described using 2-dimensional (2D) ultrasound [16]. However, the available descriptions have primarily focused on placental features such as morphology, presence of calcifications, placental lacunae, areas of thrombosis or hematomas, and anomalies in its insertion, rather than on the evaluation and quantification of size [18,19]. Therefore, only a few studies have provided data on 2D placental biometry throughout gestation, especially in the first trimester.

Imaging of the placenta has evolved rapidly in the past few years, and numerous variables estimating placental size or volume have been suggested as potential screening tools for preeclampsia or fetal growth restriction [20,21]. However, the methodology of measurement has been inconsistent in both the variable definition and in systematic measurement. Moreover, measurements have been investigated at different stages of pregnancy. Considering that the first trimester is the window of opportunity to identify pregnancies at risk for adverse outcomes, evaluating the placenta at this stage of gestation is crucial to allow for the implementation of effective prevention strategies. Finally, reference charts weighed against gestational age are necessary in order to incorporate new variables into clinical practice. These charts provide clinicians with the necessary tools to distinguish between normal and abnormal values.

The aim of this study was to construct reference ranges of 2D placental biometry and 3D PV according to gestational age and crown-rump-length (CRL) between 11 and 14 weeks of gestation.

## 2. Material and Methods

This was a prospective longitudinal study conducted at Hospital de la Santa Creu i Sant Pau between 1 September 2016 and 28 February 2020. Women with a singleton pregnancy and a fetus with a CRL between 45 and 84 mm at the time of the first trimester ultrasound were invited to participate. Exclusion criteria were: (1) pregnancy loss, (2) fetuses with chromosomal abnormalities or major fetal anomalies diagnosed during pregnancy or at birth, (3) obstetric complications such as preterm birth, preeclampsia, small-for-gestational age, placental abruptio, stillbirth or gestational diabetes, and (4) women with loss of follow-up. 

This was a planned analysis of a larger research case–control study conducted to investigate whether placental biometry, combined with maternal factors, uterine artery Doppler, blood pressure, and biochemical markers, could accurately predict the occurrence of preeclampsia and fetal growth restriction. This study was approved by the Ethics Committee of the Institutional Review Board at Hospital de la Santa Creu i Sant Pau and is registered with ClinicalTrials.gov, with number NCT02879942. 

### 2.1. Maternal and Pregnancy Characteristics

Maternal baseline characteristics were recorded including maternal age, ethnicity, body mass index (BMI), smoking habit, and medical history. Pregnancy information regarding type of conception, parity, gestational age (GA) at the time of the first trimester ultrasound, and placental location was also noted. 

### 2.2. Placental Ultrasonography

All datasets used for the analysis were acquired at the time of the first trimester ultrasound, which was conducted by experienced operators at the Prenatal Diagnosis Unit (C.T., J.P., and MC.M., A.O., O.A., named in the Acknowledgments). A single operator (C.T.) performed all of the offline measurements. To estimate placental biometry, 3D volume datasets of the placenta were acquired transabdominally using a commercially available ultrasound system (iU22 and Epiq7; Philips Healthcare, Cambridge, MA, USA). Each ultrasound system was equipped with an X6-1 Pure-Wave xMatrix transducer with an extended operating frequency range (6–1 MHz) and a 90° × 90° volume field of view. The image was adjusted to aim the placenta perpendicularly. The sweep angle was set at 90°. For each patient, two or three 3D volumes datasets were acquired to ascertain quality criteria. All volumes were scanned and saved, and images were then exported to an external hard drive for offline analysis. 

For 2D placental biometry, measurements were estimated following the methodology described by Schwartz et al. [22]. Three variables were considered to assess the 2D placental biometry: basal plate (BP), chorionic plate (CP), and placental thickness (PT), all of which were measured in two orthogonal planes as previously described [20,21]. A curvilinear measurement was used to measure BP and CP, while a linear measurement was used for PT. PT was defined as the maximal thickness observed in the image, independently of the cord insertion site. Retroplacental veins were carefully excluded for BP and PT measurements. Mean values of the two measurements were used for the analysis.

For placental volume calculation, QLAB GI3DQ software version 10.5 (Philips Healthcare; Cambridge, MA, USA) was used. This software allows for volume estimations using a modified multiplanar methodology with a fixed number of sections of the placenta. A total of 15 sections were used to retrace the placental contour and estimate the placental volume. To estimate the placental volume, the X plane was selected as the reference, and the 3D dataset was displayed until the largest view of the placenta was identified. Then, a linear axis was drawn, resulting in a diagram of 15 parallel sections perpendicular to the reference axis X. The outer contour of each placental section was manually traced, carefully excluding all structures surrounding the placenta. The PV was automatically obtained in cubic centimeters after tracing the last section. 

**Figure 1:**
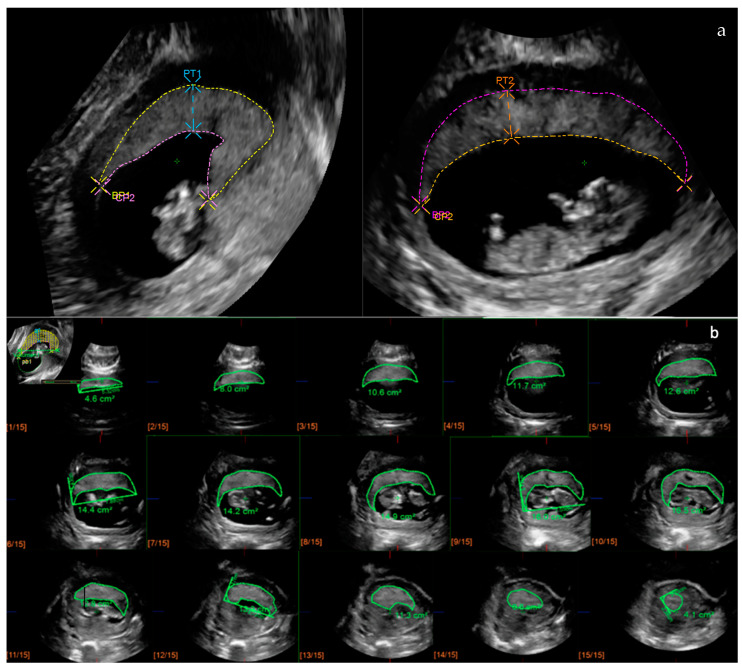
Measurement of placental biometry with 2D ultrasound and 3D ultrasound. (**a**) All variables were measured at the largest view of the placenta in two orthogonal planes. BP, basal plate; CP, chorionic plate; PT, placental thickness. (**b**) Volume calculation using QLAB GI3DQ software (15 sections). Each frame depicts the contoured area, manually traced, of the 15 sections of the placenta. PV, placental volume; 2D, three-dimensional; 3D, three-dimensional.

Figure 1 depicts the 2D and 3D measurements. Specific training for dataset acquisition and the 2D and 3D measurements was carried out prior to patient inclusion. The inter- and intraobserver agreement of these methodologies was ascertained in a previous study [23]. 

### 2.3. Statistical Analysis

For the statistical analysis, a spreadsheet format was used. The normal distribution of each variable was assessed using the Shapiro–Wilk test. In case a variable did not follow a normal distribution, a logarithmic transformation was performed as appropriate. To estimate reference ranges for 2D and 3D placental measurements according to GA, we used the statistical method described by Royston and Wright [24]. For each variable, a regression model was obtained using GA as the independent variable. For both the mean and standard deviation (SD), modeling was performed to determine which was the best model using the backward selection method. Z-scores [(measurement − mean)/(SD)] were created. The model was considered valid if these followed a normal distribution. The Shapiro–Wilk test was used to assess the normal distribution of Z-scores. For each of the variables, a table with descriptive statistics and a box plot are presented. Reference ranges (centiles 5, 10, 50, 90, 95) were calculated for BP, CP, PT, and PV based on GA (weeks) and CRL. The significance level was set at 0.05 in all tests. The analyses were conducted using SAS v9.4 software, SAS Institute Inc., Cary, NC, USA. 

## 3. Results

A total of 1491 women with a singleton pregnancy were included in the larger case–control study. Out of those, 349 were excluded for not fulfilling the inclusion criteria for the reference range analysis, leaving 1142 pregnancies without obstetric complications for final analysis. 

Baseline characteristics of the study population are shown in Table 1. Most women were Caucasian or Latin-American (91.2%), non-smokers (95.8%), and nulliparous (57.5%). In 9% of cases, pregnancy was achieved through assisted reproductive technology. The placenta was most commonly located anteriorly or posteriorly (87.7%), whereas other locations were rare. Mean GA at the time of the first-trimester ultrasound was 12 + 6 weeks’ gestation.

**Figure 2:**
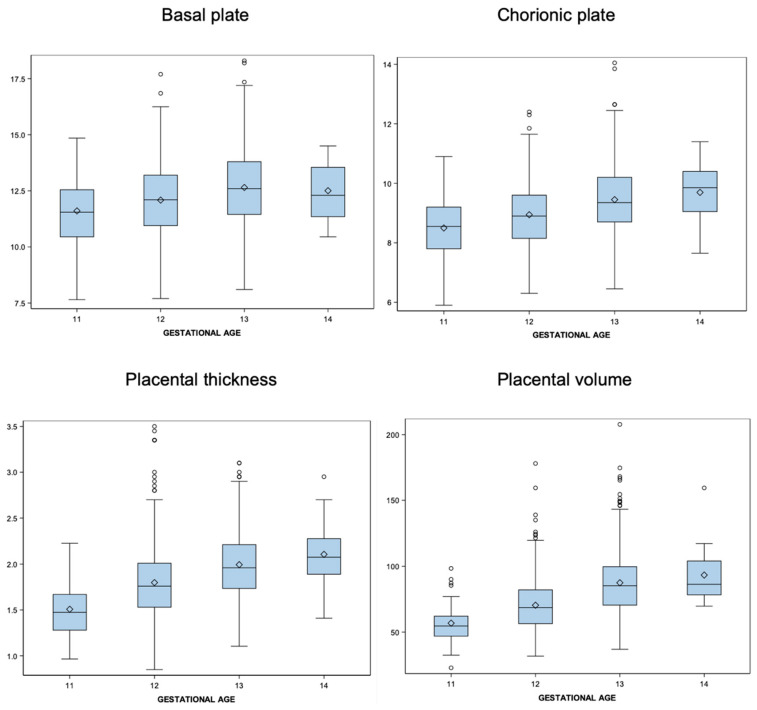
Distribution of the 2D and 3D placental biometry at each gestational week from 11 weeks to 14 weeks.

A normal distribution of both the 2D placental biometry and PV was confirmed prior to statistical analysis. The mean BP, CP, PT, and PV ranged from 11.61 cm, 8.50 cm, 1.51 cm, and 56.77 cm^3^ at 11 weeks to 12.51 cm, 9.69 cm, 2.11 cm, and 93.31 cm^3^ at 14 weeks, respectively. Placental size increased during the first trimester, showing an average percent increase of 7.75% for BP, 14% for CP, 39.64% for PT, and 64.4% for PV between 11 and 14 weeks. Statistics including the mean values and standard deviation for each placental variable are presented in Table 2. Figure 2 shows the box plots of the observed values for each 2D and 3D placental measurement against GA. Calculated reference ranges according to GA are displayed in Table 3 including the 5th, 10th, 50th, 90th and 95th percentiles. Additional reference ranges for each placental variable according to CRL are also provided in the Supplementary Materials.

## 4. Discussion

This study provides reference ranges for 2D and 3D placental biometry between 11 and 14 weeks of gestation according to GA (weeks) and CRL in singleton pregnancies. These charts were constructed including a large sample of uncomplicated pregnancies. 

Reference ranges are commonly used as a decision-making tool in clinical practice. Their main objective is to provide guidance for classifying observations within the normal or abnormal range, with the latter situation requiring additional evaluations. There are three other published studies reporting reference tables for PV in the first trimester [25,26,27]; however, these previous studies included much smaller samples of first-trimester pregnancies, with less than 50 cases per gestational week. When constructing reference ranges, the larger the sample size, the more reliable the reference ranges [28]. Considering the large number of estimations for each gestational week included in our study, we believe our results can provide more accurate reference ranges for first-trimester placental biometry.

This is the first study providing reference charts for both 2D and 3D ultrasound measurements of the placenta in the first trimester. Reproducibility of the methodology used in our study was previously assessed, showing good to excellent agreement between observers for each placental variable [23]. Assessing agreement between observers is essential, it suggests that the results are accurate and reliable. Before considering implementing any variable that could be observer-dependent into clinical practice, the methodology of measurement should be clearly defined, and the consistency of measurement across observers should also be verified. This is of great importance in the case of variables whose measurement relies, to some extent, on subjective interpretation by observers [29]. 

Although some data on PV have been published in previous research, data on 2D placental biometry in the first trimester are scarce. Our results showed that all placental measurements increased significantly with GA, especially for PT and PV. By providing results plotted against GA, we sought to provide clinicians with different tools to assess different placental biometry variables in the first trimester. The significant correlation between GA and placental measurements may offer new insights into fetal development and placental health. Our study also underscores the importance of reference charts for early placental assessment as a potential marker for pregnancy complications. These reference charts could assist clinicians in identifying placental abnormalities earlier in pregnancy, leading to improved monitoring and management strategies.

The assessment of placental biometry using 2D ultrasound is a straightforward technique to learn and perform. Schwartz et al. defined a study methodology based on the mean value of linear measurements taken in two orthogonal planes for each 2D placental variable [22]. This methodology was used for placental evaluation at 18–24 weeks, and they found a significant association of all measurements with the incidence of small-for-gestational-age fetuses. In our study, the same methodology was applied in the first trimester, incorporating a single variation: the use of a continuous, nonlinear trace for measurements. This variation is likely to provide a more accurate measurement of the placenta. 

Another study assessed the utility of 2D placental biometry in the first trimester to predict obstetric complications [27]. However, in this study, GA was determined based on the last menstrual period, adjusting for CRL only in the case of a discrepancy of more than 7 days between the last menstrual period and ultrasound dating. Considering that placental biometry measurements for screening models should be taken between 11 and 14 weeks, a nearly 7-day difference in gestational dating could have a significant impact on the interpretation of the results. Therefore, the methodology used in our study has the potential to provide more reliable results regarding the clinical utility of 2D placental biometry in predicting adverse obstetric outcomes related to placental insufficiency.

Our study has several strengths. First, these reference ranges were constructed following a well-defined methodology after the intra- and interobserver reliability of the measurement was verified [22,23]. It is crucial to verify a good level of interobserver agreement in the measurement of a variable before its clinical implementation to guarantee that different practitioners produce consistent and reliable results, thereby improving patient outcomes and the overall quality of care. Second, given the large sample size of women included, the provided reference ranges are more likely to be accurate, and thus more likely to be clinically implemented. Despite these strengths, our study also had some limitations. PV reference ranges were reported using either GA or CRL as the reference. Charts constructed using CRL have the potential to be more accurate since CRL is dependent on gestational age. However, constructing charts using CRL as the reference would require an even more significant number of cases per CRL value in order to obtain reliable results. Therefore, having weekly data may be of greater clinical utility. Second, in our study, reference ranges were constructed without customizing by maternal ethnicity, weight, or height. Customizing reference charts can be valuable in terms of external validity. However, external validity also depends on other factors such as the data quality, study design, and sample representativeness [30]. Therefore, customizing reference charts alone does not automatically guarantee higher external validity.

In summary, this study provides new reference ranges for 2D and 3D placental measurements in the first trimester. These standards were validated against pregnancy outcomes in a large sample of uncomplicated singleton pregnancies. Further studies are needed to explore the added value of 2D and 3D placental biometry to screen for placental insufficiency in the first trimester, individually or in combination with other known variables related to adverse pregnancy outcomes, as a standard practice in early prenatal care. Future research should aim to validate our results in larger, more diverse populations to enhance applicability. 

## Figures and Tables

**Table 1:** Baseline characteristics of the study population.

Characteristics	*N* = 1142
Age (years)	33.4 (5.03)
Ethnicity	
Caucasian	791 (69.3)
Latin-American	250 (21.9)
African American	23 (2.0)
Asian	28 (2.5)
South-Asian	19 (1.7)
North-African	31 (2.7)
BMI (Kg/m^2^)	24.0 (4.3)
Smoking habit	48 (4.2)
Medical history	
Chronic hypertension	10 (0.9)
Diabetes mellitus	7 (0.6)
Renal disease	3 (0.3)
Autoimmune disease	10 (0.9)
Thrombophilia	13 (1.1)
Neurologic condition	10 (0.9)
Thyroid disorders	97 (8.5)
Others	17 (1.5)
Assisted reproductive technology	103 (9.0)
Obstetric history	
Nulliparous	657 (57.5)
Parous	485 (42.5)
Placental location	
Anterior	529 (46.3)
Posterior	473 (41.4)
Lateral	93 (8.1)
Fundal	32 (2.8)
Others	3 (0.3)
First-trimester ultrasound	
Gestational age (weeks)	12.9 (0.6)
CRL (mm)	65.8 (7.9)

BMI: Body mass index; CRL: crown-rump length; data are expressed as mean (SD) or *n* (%).

**Table 2:** Descriptive statistics of the 2D and 3D placental biometry.

**Basal plate**						
Gestational age	*N*	Mean	Median	SD	Lower Quartile	Upper Quartile
11	71	11.61	11.55	1.461	10.450	12.550
12	551	12.09	12.10	1.655	10.950	13.200
13	493	12.65	12.60	1.707	11.450	13.800
14	27	12.51	12.30	1.313	11.350	13.550
**Chorionic plate**						
Gestational age	p5	p10	p50	p90	p95	
11	71	8.498	8.550	1.003	7.800	9.200
12	551	8.943	8.900	1.057	8.150	9.600
13	493	9.451	9.350	1.104	8.700	10.200
14	27	9.694	9.850	1.007	9.050	10.400
**Placental thickness**						
Gestational age	p5	p10	p50	p90	p95	
11	71	1.508	1.475	0.272	1.280	1.670
12	551	1.799	1.760	0.373	1.530	2.010
13	493	1.994	1.960	0.355	1.735	2.210
14	27	2.106	2.075	0.337	1.890	2.275
**Placental volume**						
Gestational age	p5	p10	p50	p90	p95	
11	71	56.772	54.600	13.270	46.900	62.100
12	551	70.407	68.600	19.712	56.400	82.100
13	491	87.478	85.200	23.547	70.500	99.700
14	27	93.319	86.300	19.511	78.300	104.000

**Table 3:** Reference ranges for each 2D and 3D placental measurement.

**Basal plate**					
Gestational age	p5	p10	p50	p90	p95
11	9.20	9.74	11.61	13.48	14.01
12	9.37	9.97	12.09	14.21	14.81
13	9.84	10.47	12.65	14.83	15.46
14	10.35	10.83	12.51	14.19	14.67
**Chorionic plate**					
Gestational age	p5	p10	p50	p90	p95
11	6.89	7.21	8.50	9.78	10.15
12	7.20	7.59	8.94	10.30	10.68
13	7.63	8.04	9.45	10.86	11.27
14	8.04	8.41	9.69	10.98	11.35
**Placental thickness**					
Gestational age	p5	p10	p50	p90	p95
11	1.06	1.16	1.51	1.86	1.96
12	1.19	1.32	1.80	2.28	2.41
13	1.41	1.54	1.99	2.45	2.58
14	1.55	1.67	2.11	2.54	2.66
**Placental volume**					
Gestational age	p5	p10	p50	p90	p95
11	34.94	39.79	56.78	73.76	78.60
12	37.98	45.18	70.41	95.64	102.83
13	48.74	57.34	87.48	117.62	126.21
14	61.22	68.34	93.32	118.29	125.41

## Data Availability

The data presented in this study are available on request from the corresponding author.

## Supplementary Materials

The following supporting information can be downloaded at: https://www.mdpi.com/article/10.3390/diagnostics14141556/s1, Table S1. Reference ranges for 2D basal plate measurements according to crown-rump-length (CRL). Table S2. Reference ranges for 2D chorionic plate measurements according to crown-rump-length (CRL). Table S3. Reference ranges for 2D placental thickness measurements according to crown-rump-length (CRL). Table S4. Reference ranges for 3D placental volume measurements according to crown-rump-length (CRL).
