# Supplementary material for: Reference Ranges of 2-Dimensional Placental Biometry and 3-Dimensional Placental Volume between 11 and 14 Weeks of Gestation
Source: Diagnostics (Basel). 2024 Jul 18;14(14):1556. doi: 10.3390/diagnostics14141556 (PMC11276113; doi:10.3390/diagnostics14141556)
Supplement: Supplementary file 1 [file diagnostics-14-01556-s001.zip › diagnostics-3084524-supplementary.pdf]

Table S1. Reference ranges for 2D basal plate measurements according to crown-rump-length (CRL).

CRL	p5	p10	p50	p90	p95
45.0	8.10	8.62	11.35	14.08	14.60
46.0	8.15	8.67	11.40	14.12	14.65
47.0	8.20	8.72	11.44	14.17	14.69
48.0	8.24	8.76	11.49	14.21	14.74
49.0	8.29	8.81	11.54	14.26	14.78
50.0	8.33	8.86	11.58	14.31	14.89
51.0	8.38	8.91	11.63	14.35	14.87
52.0	8.43	8.95	11.67	14.40	14.92
53.0	8.48	8.99	11.72	14.44	14.96
54.0	8.52	9.05	11.77	14.49	15.01
55.0	8.57	9.09	11.81	14.53	15.06
56.0	8.62	9.14	11.86	14.58	15.10
57.0	8.66	9.18	11.91	14.62	15.14
58.0	8.71	9.23	11.95	14.67	15.19
59.0	8.75	9.27	11.99	14.72	15.24
60.0	8.80	9.32	12.04	14.76	15.28
61.0	8.85	9.37	12.09	14.81	15.33
62.0	8.89	9.42	12.14	14.85	15.38
63.0	8.94	9.46	12.18	14.90	15.42
64.0	8.99	9.51	12.23	14.95	15.47
65.0	9.03	9.55	12.27	14.99	15.51
66.0	9.08	9.60	12.32	15.04	15.56
67.0	9.13	9.6485	12.3671	15.0857	15.6073
68.0	9.17296	9.6946	12.4133	15.1319	15.6536
69.0	9.21898	9.7406	12.4594	15.1782	15.6998
70.0	9.26494	9.7866	12.5055	15.2245	15.7461

CRL	p5	p10	p50	p90	p95
71.0	9.31	9.83	12.55	15.27	15.79
72.0	9.34	9.88	12.60	15.32	15.84
73.0	9.40	9.92	12.64	15.36	15.88
74.0	9.45	9.97	12.69	15.41	15.93
75.0	9.49	10.02	12.73	15.46	15.98
76.0	9.54	10.06	12.78	15.50	16.02
77.0	9.58	10.10	12.83	15.55	16.07
78.0	9.63	10.15	12.87	15.59	16.12
79.0	9.68	10.20	12.92	15.64	16.16
80.0	9.72	10.24	12.97	15.69	16.21
81.0	9.77	10.29	13.01	15.74	16.26
82.0	9.81	10.33	13.06	15.78	16.30
83.0	9.86	10.38	13.10	15.83	16.35
84.0	9.90	10.43	13.15	15.88	16.40
85.0	9.95	10.47	13.20	15.92	16.44

Table S2. Reference ranges for 2D chorionic plate measurements according to crown-rump-length (CRL).

CRL	p5	p10	p50	p90	p95
45.0	6.13	6.46	8.20	9.95	10.28
46.0	6.18	6.51	8.25	9.9910	10.32
47.0	6.22	6.55	8.29	10.04	10.37
48.0	6.27	6.60	8.34	10.08	10.41
49.0	6.31	6.65	8.39	10.13	10.46
50.0	6.36	6.69	8.43	10.17	10.50
51.0	6.41	6.74	8.48	10.22	10.55
52.0	6.45	6.79	8.52	10.26	10.59
53.0	6.50	6.83	8.57	10.31	10.64
54.0	6.54	6.88	8.61	10.35	10.68
55.0	6.59	6.92	8.67	10.40	10.73
56.0	6.64	6.97	8.71	10.44	10.78
57.0	6.68	7.01	8.75	10.49	10.82

CRL	p5	p10	p50	p90	p95
58.0	6.73	7.06	8.79	10.53	10.87
59.0	6.77	7.11	8.84	10.58	10.91
60.0	6.82	7.15	8.89	10.62	10.96
61.0	6.86	7.20	8.93	10.67	11.00
62.0	6.91	7.24	8.98	10.71	11.05
63.0	6.96	7.29	9.02	10.76	11.09
64.0	7.00	7.33	9.07	10.80	11.13
65.0	7.05	7.38	9.11	10.85	11.18
66.0	7.09	7.43	9.16	10.89	11.23
67.0	7.14	7.47	9.21	10.94	11.27
68.0	7.18	7.52	9.25	10.99	11.32
69.0	7.23	7.56	9.29	11.03	11.67
70.0	7.27	7.61	9.34	11.08	11.41
71.0	7.32	7.65	9.39	11.12	11.46
72.0	7.36	7.69	9.43	11.17	11.50
73.0	7.41	7.74	9.48	11.22	11.55
74.0	7.46	7.79	9.52	11.26	11.59
75.0	7.50	7.83	9.57	11.31	11.64
76.0	7.55	7.88	9.62	11.35	11.69
77.0	7.59	7.92	9.66	11.40	11.73
78.0	7.63	7.97	9.71	11.44	11.78
79.0	7.68	8.02	9.75	11.49	11.82
80.0	7.73	8.06	9.80	11.54	11.87
81.0	7.77	8.11	9.84	11.58	11.91
82.0	7.82	8.15	9.89	11.63	11.96
83.0	7.86	8.20	9.93	11.67	12.01
84.0	7.91	8.24	9.98	11.72	12.05
85.0	7.95	8.29	10.03	11.77	12.10

Table S3. Reference ranges for 2D placental thickness measurements according to crown-rump-length (CRL).

CRL	P5	P10	p50	P90	P95
45.0	0.796	0.898	1.484	2.069	2.181
46.0	0.805	0.917	1.502	2.088	2.200
47.0	0.824	0.936	1.521	2.106	2.218
48.0	0.843	0.955	1.540	2.125	2.237
49.0	0.862	0.974	1.559	2.143	2.255
50.0	0.880	0.993	1.577	2.162	2.274
51.0	0.899	1.011	1.596	2.180	2.293
52.0	0.918	1.030	1.615	2.199	2.311
53.0	0.937	1.049	1.633	2.218	2.329
54.0	0.956	1.068	1.652	2.236	2.348
55.0	0.975	1.087	1.671	2.255	2.367
56.0	0.993	1.105	1.689	2.273	2.386
57.0	1.012	1.124	1.708	2.292	2.404
58.0	1.031	1.143	1.727	2.311	2.423
59.0	1.050	1.162	1.746	2.330	2.441
60.0	1.068	1.180	1.764	2.348	2.460
61.0	1.087	1.199	1.783	2.367	2.479
62.0	1.106	1.218	1.802	2.385	2.497
63.0	1.125	1.237	1.820	2.404	2.516
64.0	1.143	1.255	1.839	2.423	2.535
65.0	1.162	1.274	1.858	2.441	2.553
66.0	1.181	1.293	1.876	2.460	2.572
67.0	1.199	1.311	1.895	2.479	2.591
68.0	1.218	1.330	1.914	2.497	2.609
69.0	1.237	1.349	1.932	2.516	2.628
70.0	1.255	1.367	1.951	2.535	2.647
71.0	1.274	1.386	1.970	2.554	2.665
72.0	1.293	1.405	1.989	2.572	2.684

CRL	P5	P10	p50	P90	P95
73.0	1.311	1.423	2.007	2.591	2.703
74.0	1.330	1.442	2.026	2.610	2.722
75.0	1.349	1.461	2.045	2.629	2.741
76.0	1.367	1.479	2.063	2.647	2.759
77.0	1.386	1.498	2.082	2.666	2.778
78.0	1.404	1.516	2.101	2.685	2.797
79.0	1.423	1.535	2.119	2.704	2.816
80.0	1.442	1.554	2.138	2.723	2.835
81.0	1.460	1.572	2.157	2.741	2.854
82.0	1.479	1.591	2.176	2.760	2.873
83.0	1.497	1.609	2.194	2.779	2.891
84.0	1.516	1.628	2.213	2.798	2.910
85.0	1.534	1.646	2.232	2.817	2.929

Table S4. Reference ranges for 3D placental volume measurements according to crown-rump-length (CRL).

CRL	p5	p10	p50	p90	p95
45.0	7.21	13.68	47.34	81.01	87.47
46.0	8.68	15.14	48.79	82.45	88.91
47.0	10.14	16.59	50.24	83.89	90.34
48.0	11.60	18.05	51.69	85.33	91.79
49.0	13.06	19.51	53.14	86.77	93.23
50.0	14.52	20.97	54.59	88.22	94.67
51.0	15.98	22.43	56.04	89.66	96.11
52.0	17.43	23.88	57.49	91.10	97.55
53.0	18.89	25.34	58.94	92.54	98.99
54.0	20.35	26.80	60.39	93.99	100.44
55.0	21.81	28.25	61.84	95.43	101.88
56.0	23.26	29.71	63.29	96.88	103.32
57.0	24.72	31.16	64.74	98.32	104.77
58.0	26.17	32.61	66.19	99.77	106.21
59.0	27.63	34.07	67.64	101.22	107.66

CRL	p5	p10	p50	p90	p95
60.0	29.08	35.52	69.09	102.66	109.11
61.0	30.53	36.97	70.54	104.11	110.55
62.0	31.98	38.42	71.99	105.56	112.00
63.0	33.44	39.88	73.44	107.01	113.48
64.0	34.89	41.33	74.89	108.46	114.90
65.0	36.34	42.78	76.34	109.91	116.35
66.0	37.79	44.23	77.79	111.36	117.80
67.0	39.24	45.68	79.24	112.81	119.25
68.0	40.69	47.13	80.69	114.26	120.70
69.0	42.13	48.58	82.14	115.71	122.15
70.0	43.58	50.02	83.59	117.16	123.60
71.0	45.03	51.47	85.04	118.61	125.05
72.0	46.48	52.92	86.49	120.06	126.51
73.0	47.92	54.36	87.94	121.52	127.96
74.0	49.37	55.81	89.39	122.97	129.41
75.0	50.81	57.26	90.84	124.43	130.87
76.0	52.26	58.70	92.29	125.88	132.32
77.0	53.70	60.15	93.74	127.34	133.78
78.0	55.14	61.59	95.19	128.79	135.24
79.0	56.59	63.04	96.64	130.25	136.69
80.0	58.03	64.48	98.09	131.70	138.15
81.0	59.47	65.92	99.54	133.16	139.61
82.0	60.91	67.36	100.99	134.62	141.07
83.0	62.35	68.81	102.44	136.08	142.53
84.0	63.79	70.25	103.89	137.53	143.99
85.0	65.23	71.69	105.34	138.99	145.45
